# Trade-Marks and Copy-Rights

**Published:** 1882-04-20

**Authors:** R. L. Hinton

**Affiliations:** Ky.; Arkansas


### TRADE-MARKS AND COPY-RIGHTS.

BY R. L. HINTON, M. D., OF ARKANSAS.

*Messrs. Editors Southern Medical Record:*

"A moment's reflection must convince many physicians that the interests of the medical profession are seriously hazarded when physicians consent to prescribe and promote the general use of every proprietary pharmaceutical candidate armed and helmetted with trade-mark and copy-right exclusiveness that forces itself into their presence—while the immense resources of the open materia medica are always at hand to meet every indication of disease."

I take the above extract verbatim, from an article in the *Pharmaceutical Gazette*, by George B. Swayze, M. D., of Philadelphia, (rather from the *Therapeutic Gazette*, Nov. No.), which strikes me very forcibly, from the fact that I have so frequently met with physicians, who are prescribing and using these patent nostrums in their practice, recommending them to me, and seemed to be even surprised that I was not using them—supposing, of course, that I am behind the times or not fully posted, to say the least. Well, I may not be fully posted, but I have this to say—that if a physician cannot meet the indications of disease with a judicious selection and combination of the remedies found in our materia medica, with whose properties he ought to be well acquainted, he certainly is going it blind, when he prescribes a compound, not knowing himself of what it is composed, or what to expect from its use, further than what he reads on the label, or in an almanac. The responsibility of a physician is, in my estimation, too great, to be shifted off in this way.

